# Laser Therapy Effects on Periodontal Status: A Randomized Study Using Gaussian Network Analysis and Structural Equation Modeling Approach

**DOI:** 10.3390/medicina60030437

**Published:** 2024-03-06

**Authors:** Codruta Elena Ciurescu, Lorena Dima, Vlad Alexandru Ciurescu, Gratiela Georgiana Noja, Alin Viorel Istodor, Marius Alexandru Moga, Lavinia Cosmina Ardelean, Laura-Cristina Rusu, Marius Traian Leretter

**Affiliations:** 1 Department of Fundamental Disciplines and Clinical Prevention, Faculty of Medicine Brasov, “Transylvania” University of Brasov, 56 Nicolae Balcescu Str., 500019 Brasov, Romania; codruta.chira@unitbv.ro (C.E.C.); lorena.dima@unitbv.ro (L.D.); 2 Faculty of Dental Medicine, “Iuliu Hatieganu” University of Medicine and Pharmacy, 8 Victor Babes Str., 400012 Cluj-Napoca, Romania; ciurescuv@yahoo.com; 3 Department of Marketing and International Economic Relations, Faculty of Economics and Business Administration, West University of Timisoara, 16 Pestalozzi Str., 300115 Timisoara, Romania; gratiela.noja@e-uvt.ro; 4 First Department of Surgery, Second Discipline of Surgical Semiology, “Victor Babes” University of Medicine and Pharmacy Timisoara, 2 Eftimie Murgu Sq., 300041 Timisoara, Romania; istodor.alin@umft.ro; 5 Department of Medical and Surgical Specialities, Faculty of Medicine, “Transylvania” University of Brasov, 56 Nicolae Balcescu Str., 500019 Brasov, Romania; mogas@unitbv.ro; 6 Department of Technology of Materials and Devices in Dental Medicine, Multidisciplinary Center for Research, Evaluation, Diagnosis and Therapies in Oral Medicine, “Victor Babes” University of Medicine and Pharmacy Timisoara, 2 Eftimie Murgu Sq., 300041 Timisoara, Romania; 7 Department of Oral Pathology, Multidisciplinary Center for Research, Evaluation, Diagnosis and Therapies in Oral Medicine, “Victor Babes” University of Medicine and Pharmacy Timisoara, 2 Eftimie Murgu Sq., 300041 Timisoara, Romania; laura.rusu@umft.ro; 8 Department of Prosthodontics, Multidisciplinary Center for Research, Evaluation, Diagnosis and Therapies in Oral Medicine, “Victor Babes” University of Medicine and Pharmacy Timisoara, 2 Eftimie Murgu Sq., 300041 Timisoara, Romania; mariusleretter@yahoo.com

**Keywords:** periodontitis, laser therapy, Er,Cr:YSGG, Gaussian graphical models, structural equation modeling, network analysis, non-surgical periodontal therapy, periodontal pocket depth, clinical attachment level, bleeding on probing

## Abstract

*Background and Objectives:* This paper aims to assess the role of laser therapy in periodontitis through an innovative approach involving computational prediction and advanced modeling performed through network analysis (Gaussian graphical models—GGMs) and structural equations (SEM). *Materials and Methods:* Forty patients, exhibiting periodontal pockets with a minimum depth of 5 mm, were randomly divided into two groups: a control group and a laser group. Four specific indicators were measured for each tooth, namely periodontal pocket depth (PPD), clinical attachment level (CAL), bleeding on probing (BOP), and plaque index (PI), and the mean of six measured values was recorded at five time markers (baseline, 6 months, 1 year, 2 years, and 4 years). The assessment algorithm included enrollment, measurements, and differential non-surgical periodontal treatment, according to the group allocation. Scaling, root planing, and chlorhexidine 1% were conducted for the control group, and scaling, root planing and erbium, chromium:yttrium-scandium-gallium-garnet (Er,CR:YSGG) laser therapy were conducted for the laser group. *Results:* The main results highlight that the addition of laser treatment to scaling and root planing led to notable clinical improvements, decreasing the PPD values, reducing the BOP scores, and increasing the CAL. *Conclusions:* Notable relationships between the specific indicators considered were highlighted by both the GGMs and by SEM, thus confirming their suitability as proxies for the success of periodontal treatment.

## 1. Introduction

Laser therapy was gradually introduced in dentistry in the early 1990s and has since successfully broadened its applications. The use of lasers is considered suitable and effective for treating a variety of inflammatory and infectious conditions [1] and has been utilized in the treatment of periodontitis or peri-implantitis since the early 2000s.

Periodontal disease is commonly defined as an inflammatory disorder involving both soft and hard periodontal structures, with multifactorial causes, and it is strongly associated with numerous general pathologies [2].

However, its main triggering factor is dental plaque and the subsequent inflammatory reaction of the subject. It has a progressive evolution and, when left untreated, usually results in tooth loss. The prevalence of periodontal disease was reported to range from 20% to 50% worldwide, and it is currently considered a global public health problem [3].

According to the 2017 World Workshop on the Classification of Periodontal and Peri-Implant Diseases and Conditions, stage 1 is characterized by the presence of dysbiosis, bleeding on probing, and the development of attachment loss.

Stage 2 is characterized by the presence of attachment loss, bleeding on probing, and early bone loss, while stages 3 and 4 involve significant bone loss.

Stage 2, as well as stages 3 and 4, require primarily non-surgical treatment, in order to arrest the inflammatory process, which involves scaling and root planing, boosted by local or systemic anti-biotherapy. In more severe cases, usually categorized as stage 4 and sometimes stage 3, surgical treatment is needed, consisting primarily of debridement and root planing. Due to the limited effects of antibiotics, and considering the risk of developing resistance, the use of laser therapy as a treatment option in controlling dysbiosis and local inflammation has been attempted [4].

The most common indicators used to categorize, assess, and monitor periodontal status include the clinical attachment level (CAL), the periodontal probing depth (PPD) measurements, and the presence of bleeding on probing (BOP) [5].

According to previous studies, laser therapy demonstrated better outcomes compared to conventional non-surgical periodontal treatment [6,7].

Erbium, chromium:yttrium-scandium-gallium-garnet (Er,Cr:YSGG) laser, a member of the erbium laser family, is considered as an effective modality for the treatment of moderate to advanced periodontal diseases [8]. It proved to be effective in ablating both soft and hard tissues [9].

Compared with other lasers, Er,Cr:YSGG lasers are characterized by shallow tissue penetration, which results in minimal thermal risk to the deeper tissues [10]. They provide a quality root surface for the attachment of blood-derived components, promoting cell migration and attachment [11], bacterial reduction, the coagulation of the opened blood vessels, and the de-epithelialization of the gingival pocket, with subsequent significant improvements in PPD and CAL [12,13,14,15].

Computational prediction and advanced modeling are modern methodological techniques, based on Gaussian graphical models (GGMs) network analysis and structural equation modeling (SEM). GGMs are exploratory research tools that are extremely useful in deducing the connections between different types of variables. A GGM is a “graph in which all random variables are continuous and jointly Gaussian” [16,17], based on conditional independence. GGMs allow for the capture of “conditional associations and avoid spurious correlation, grasping an undirected network of partial correlation coefficients (both positive visualized with blue edges and inverse captured with red edges), graphically reflected through the absolute strengths, width and saturation of the edges between nodes” [18].

SEM is a complex technique that complements GGMs, designed to model longitudinal data, which enables us “to identify and evaluate direct and latent interlinkages between several specific variables” [18]. It examines the causal relationship between variables, capturing the importance of a certain cause to effect influence, and allowing multiple causal associations between the variables [18,19].

## 2. Background of the Study

The role of laser therapy in periodontitis has been largely approached in the relevant scientific literature. To better document the background of our research, a systematic review of the literature and a bibliometric analysis of over 1500 scientific articles extracted from PubMed and Web of Science were performed. The methodological rationale of focusing on extracting the newest, most relevant, and highly cited papers published on this research topic during 2010–2023 was to capture the main research directions, updated guidelines and theoretical groundings.

**Figure 1:**
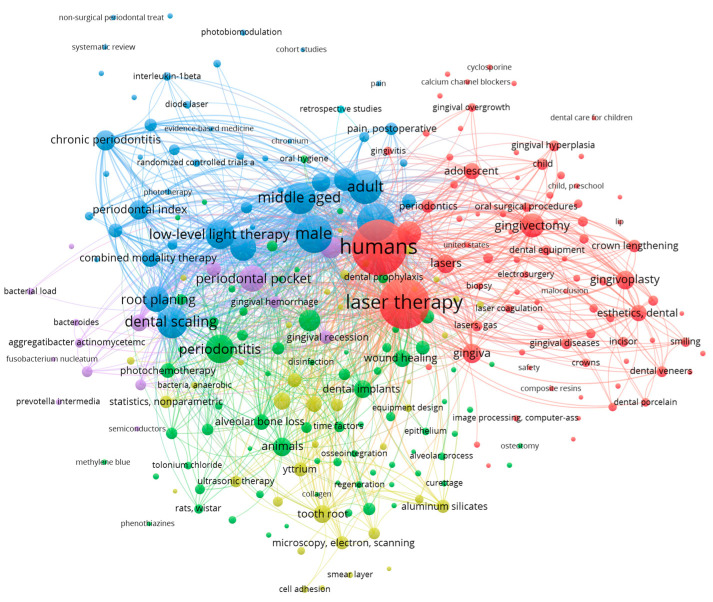
Mapping the co-occurrence of relevant scientific keywords and research directions based on PubMed data (at least 5 appearances of each keyword in the sample of articles).

**Figure 2:**
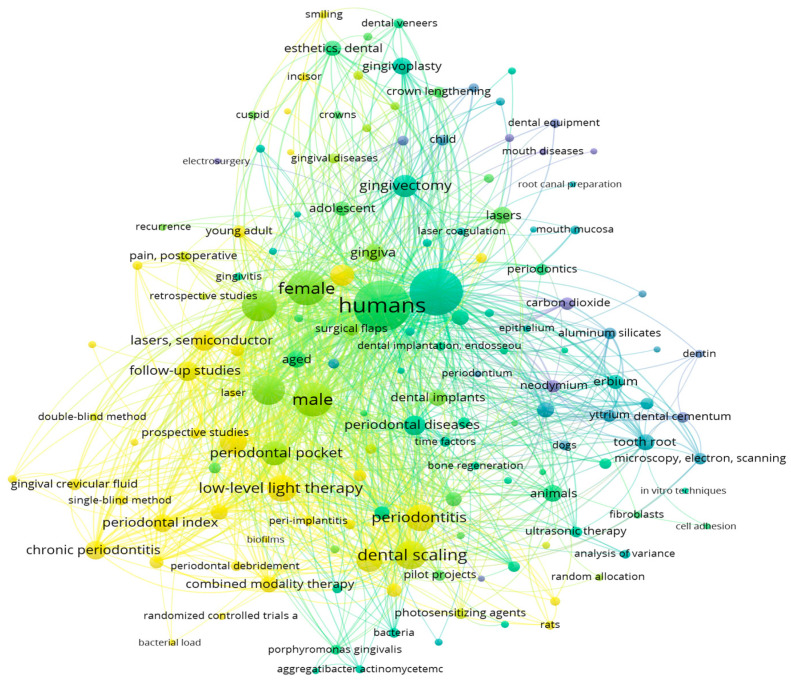
Mapping the co-occurrence of relevant scientific keywords and research directions based on PubMed data (at least 10 appearances of each keyword in the sample of articles).

**Figure 3:**
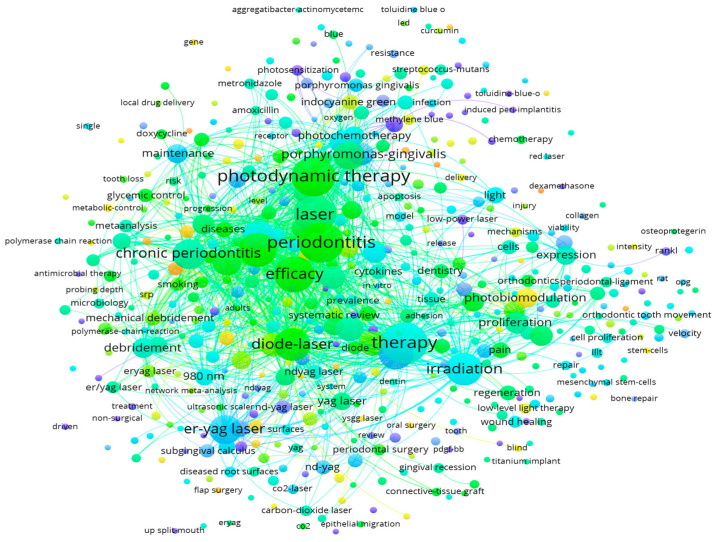
Mapping the co-occurrence of relevant scientific keywords and research directions based on Web of Science data (at least 5 appearances of each keyword in the sample of articles).

**Figure 4:**
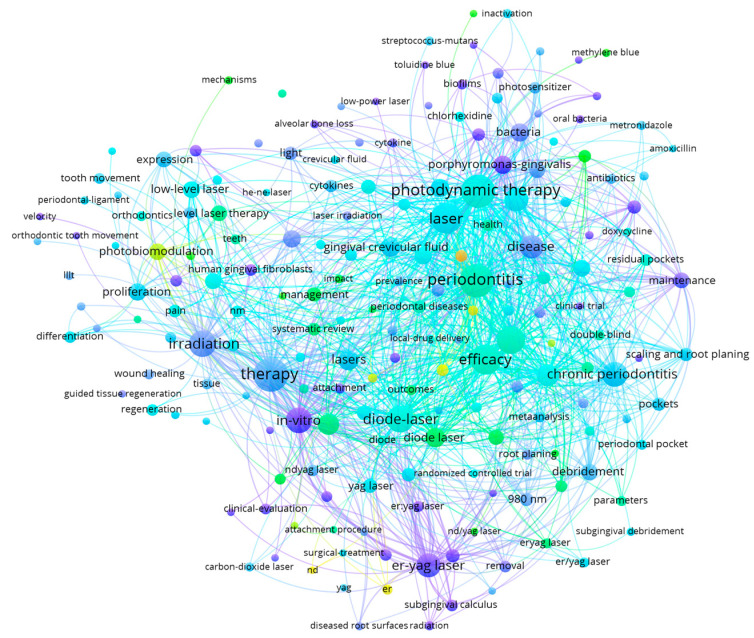
Mapping the co-occurrence of relevant scientific keywords and research directions based on Web of Science data (at least 10 appearances of each keyword in the sample of articles).

The bibliometric search was carried out using the following keywords, in line with the purpose of our research, namely: periodontal laser therapy OR laser periodontal treatment AND Er,Cr:YSGG AND computational modeling AND prediction. Based on these keywords and the criteria previously mentioned, an initial search was performed in PubMed and Web of Science databases. The final query returned a sample of 681 scientific articles in PubMed and 947 articles in Web of Science. The large amount of information extracted from these 1628 articles was further processed in VOSviewer. The main results of the analysis of the co-occurrence of relevant terms and research concepts performed on the PubMed and Web of Science article samples are graphically mapped in Figure 1, Figure 2, Figure 3 and Figure 4.

The co-occurrences of essential keywords in the analyzed sample of 1628 articles extracted from PubMed and Web of Science indicated that diode, Er:YAG, Nd:YAG, carbon-dioxide laser therapy and photodynamic therapy are usually considered by researchers in treating periodontitis. The periodontal pocket is generally measured and considered a proxy for the success of laser therapy. A notable focus is also placed on bleeding, wound healing and tissue regeneration.

The results of the performed bibliometric analysis were further used to design the general framework of the computation modeling, by means of network analysis through Gaussian graphical models (GGM) and structural equation modeling (SEM).

Considering the above-stated theoretical groundings, the research conducted in this paper aims to assess the role of laser therapy in treating periodontitis, to evaluate its impact and benefits, through an innovative comprehensive approach that embeds the nonsurgical periodontal treatment with advanced empirical modeling performed through network analysis (Gaussian graphical models—GGMs) and structural equation modeling (SEM). The advanced software and computation modeling allow researchers to delve deeper into the role and effects of laser in the therapy of periodontal disease, thus revealing key coordinates/indicators/measurements that are essential for its success.

## 3. Materials and Methods

### 3.1. Sample Description

The selection of the patients was carried out between January 2018 and June 2018. The randomized clinical study was performed during a 4-year timeframe, starting July 2018. All participants signed an informed consent before inclusion in the study. The study was conducted in accordance with the Helsinki Declaration, and the study protocol was approved by the Ethics Committee of “Transylvania” University of Brasov and “Krondent” Dental Clinic (Project identification code 101E).

Inclusion criteria:

- at least 4 dental units distributed in at least 2 quadrants present
- at least 2 periodontal pockets with a PPD ≥ 5 mm, located in 2 different quadrants

Exclusion criteria:

- patients who took antibiotics within the last 3 months or currently under antibiotics
- patients who received periodontal treatment within the last 12 months
- patients with over-contoured prosthetic restorations that do not allow proper measurements
- pregnant or lactating women
- patients with systemic diseases that may influence the treatment outcome: diabetes, cardiovascular disease, cancer, psychiatric diseases, immune diseases
- patients undergoing radiotherapy in cervical/oral area, immune therapy, taking psychiatric medication

Orthopantomogram X-rays were performed as part of the initial examination. The patients exhibiting severe bone loss, revealed by X-ray, diagnosed as stage-4 periodontitis and requiring surgical treatment, were excluded at this point.

The initial sample, consisting of 42 patients (20 males and 22 females), age 25–75 years, was randomly divided into 2 groups: control—Group 1 (CHX) and experimental—Group 2 (laser). A block randomization protocol was used. As 2 subjects belonging to Group 2 did not participate in the initial evaluation determining baseline values (T1), Group 1 consisted of 21 subjects and Group 2 of 19 subjects. The group characteristics are given in Table 1.

### 3.2. Assessment and Treatment Protocol

The dataset comprised 120 measurements for each tooth of the subjects, collected at 5 different time markers: T1—baseline, T2—6 months, T3—12 months, T4—24 months, T5—48 months.

The measurements were identically performed for both groups, using a PCP (Columbia 15 mm periodontal probe, Hu-Friedy Manufacturing Co., Chicago, IL, USA), by a dentist with extensive experience in periodontal screening. PPD was measured in mm, from the gingival margin to the most apical point of the sulcus/pocket, in 6 points for each tooth, at the nearest millimeter: mesiobuccal, buccal, distobuccal, mesiooral, oral and distooral. The gingival margin was also measured in the same points, and CAL was calculated in all sites. BOP was determined 10 s after probing and considered positive or negative according to the presence or absence of blood at the probed site. PI was evaluated by visual inspection and coded based on the Silness–Loe index (0–3).

The measurements of all parameters were repeated in the same manner at T2–T5 time markers.

The treatment algorithm, characteristic for each of the 2 groups, comprised the following:

Group 1 (CHX) control: conventional non-surgical treatment (SRP): ultrasound and manual scaling, root planing using various Gracey curettes (Hu-Friedy Manufacturing Co., Chicago, IL, USA), depending on the location and depth of the pocket, and Clorhexidine (CHX) 1% gel Curasept (Curaden, Kriens, Switzerland). The gel was injected into all pockets exhibiting a PPD ≥ 5 mm, by means of a syringe, with an attached bunt disposable plastic applicator.

Group 2 (laser): conventional non-surgical treatment (SRP): ultrasound ± manual scaling, and root planing using Gracey curettes (Hu-Friedy Manufacturing Co., Chicago, IL, USA), and, in addition, Er,Cr:YSGG laser treatment, using a Waterlase iPlus (Biolase Inc., Foothill Ranch, CA, USA), of all sites which exhibited PPD ≥ 5 mm. The laser parameters used are given in Table 2.

Treatment was performed by “painting” the respective surface in non-contact mode with a hand speed of 10 s/mm in monorooted and 15 s/mm in multirooted teeth, due to the wider diameter of the latter. The tip was placed at the bottom of the periodontal pocket, retracting it by 1 mm and then firing the laser, drawing the root surface and then the inside wall of the periodontal pocket. The treatment was completed by de-epithelization of the gingival margin and of the external side of the periodontal pocket for at least 2 mm.

The differential treatment sessions were carried out every 6 months, according to group allocation, the patients being submitted to the same treatment at each time point.

In order to achieve periodontal health, the following treatment steps were performed during this study:

At T1 (baseline), all periodontal pockets with PPD ≥ 5 mm were treated according to the protocol and groups they belonged to.

At T2, periodontal measurements were performed. If the new measured value of PPD was below 5 mm, periodontal treatment was no longer required. The sites exhibiting PPD values ≥ 5 mm were further submitted to periodontal treatment, according to their group.

At T3 and T4, consequent periodontal measurements were performed, and the same protocol was applied: the sites exhibiting values less than 5 mm did not receive further periodontal treatment.

At T5, the final periodontal measurements were performed, and the final results were collected.

The patients who showed no PPD values ≥ 5 mm at a certain time point were no longer assessed, as they did not need further periodontal treatment, being declared healed at that time point. The number of patients belonging to each group, at the different time markers, are shown in Table 3.

### 3.3. Computational Analysis

The computational prediction and advanced modeling were performed through network analysis (Gaussian graphical models—GGMs) and structural equations (SEM), based on the measurements collected at T1–T5 time markers. The indicators considered in this investigation were PPD, CAL, BOP and PI.

Both GGMs and SEM provide robust estimates since they test multiple associations in a single model and capture the inferences and interlinkages among all considered indicators.

Two GGMs models were configured and estimated through two distinctive methods, namely the extended Bayesian information criteria with graphical least absolute shrinkage and selection operator (EBICglasso) and partial correlation (PCOR). SEM aimed at capturing the periodontal health status, reflected and resulting from the indicator measurements, as a latent variable (PH, PH_L), highlighting the patterns and correlations between indicators and identifying and evaluating the causal relationship.

### 3.4. Statistical Analysis

The measured values for PPD, CAL, BOP and PI, for both groups, at T1–T5 time frames, were stored as Excel files, and the mean + SD values were calculated.

The periodontal pockets were divided into three depth groups, based on PPD measurements at T1, namely 4–5 mm, 6–7 mm, and ≤8 mm, and followed in dynamics until T5. Each pocket’s depth decreased following treatment and consequently was moved to an inferior depth group, being possibly or eventually declared healed and no longer requiring treatment. 

Data analysis was carried out with Python 3.0 software (Python Software Foundation, Wilmington, DE, USA).

The *t*-test was used to evaluate and compare the mean values of the indicators recorded for each of the two groups, and *p* was used to determine if the differences were the result of a normal variability or indicated a significant impact or difference between groups. *p* < 0.05 was considered statistically significant.

## 4. Results

### 4.1. Indicator Measurements

Each indicator, namely PPD, CAL, BOP, and PI, was measured for each tooth, at T1–T5 time markers. The collected data and the *p* values are shown in Table 4, with the * indicating statistically significant *p* values (*p* < 0.05). The borderline statistically significant *p* values (*p* = 0.05), suggesting a marginal significance, are marked with ^†^.

**Figure 5:**
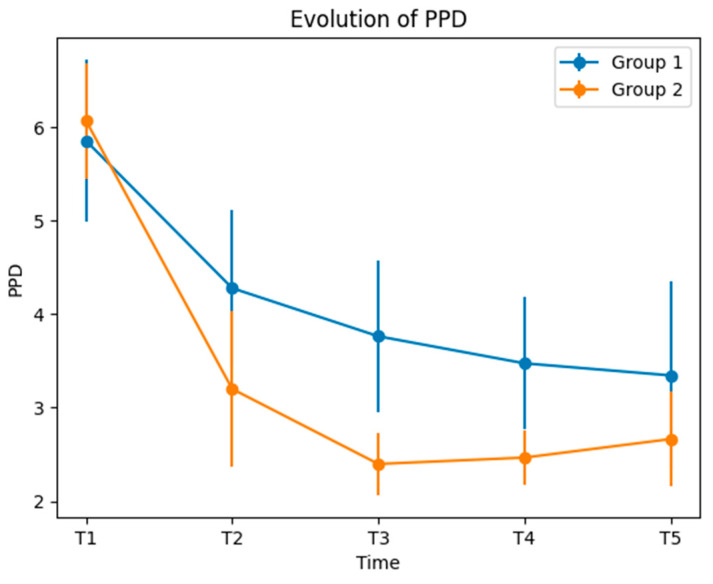
Evolution of PPD.

At T1, the PPD mean values showed no evidence of significant differences between the groups. At T2, there was a significantly different evolution of PPD. Group 2 experimenting a better treatment outcome. At T3, the PPD values for Group 2 as well were significantly lower compared to Group 1. This trend continued for T4 and T5. PPD showed significant lower values in Group 2 compared to Group 1, at all T2–T5 time markers (Table 4 and Figure 5).

The number of PPD sites, grouped by depth, at T1–T5 is shown in Table 5.

In the case of CAL, at T1 (baseline) and T2, there was no significant difference between the groups. However, the differences between CAL values at T1 and T2 were statistically significant (*p* = 0.007), which indicates an important difference between the groups, the attachment gain being higher for Group 2 compared to Group 1.

**Figure 6:**
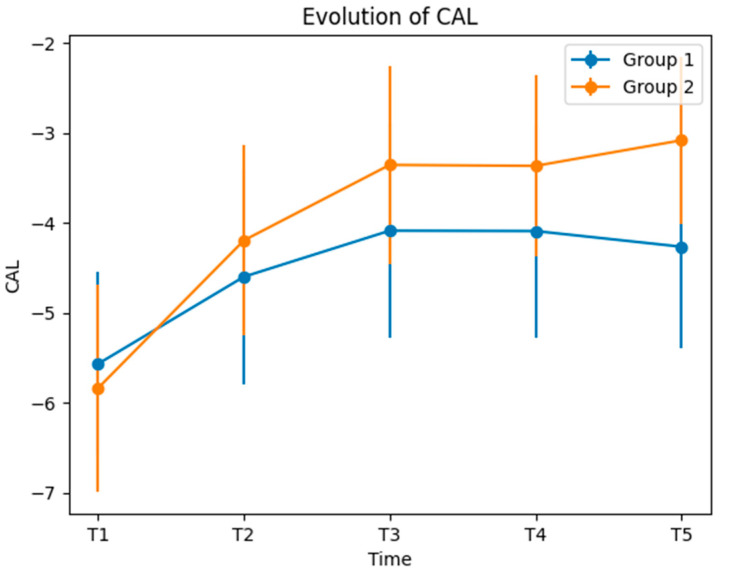
Evolution of CAL.

At T3, Group 2 experienced a significant change compared to Group 1, this issue being also reflected in the difference between the T1–T3 mean + SD values. At T4 and T5, the mean + SD CAL values for Group 1 were, once again, higher than those of Group 2, having statistical significance and indicating that Group 2 developed a more extensive transformation than Group 1 (Table 4 and Figure 6).

**Figure 7:**
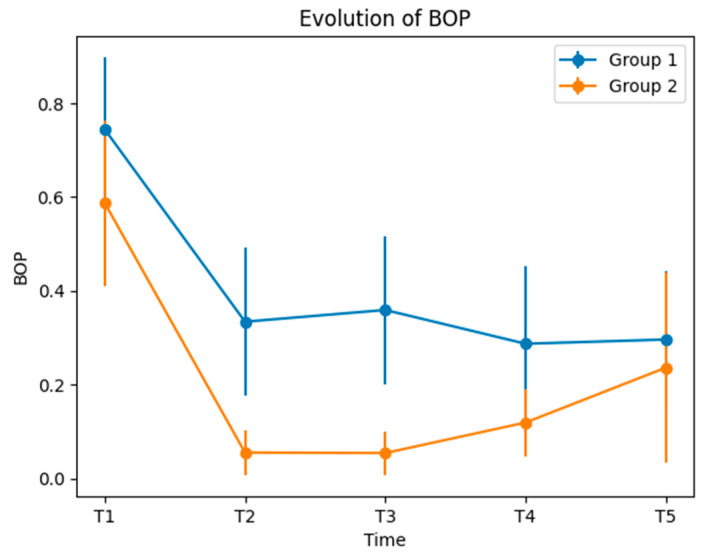
Evolution of BOP.

BOP values at T1 were significantly higher in Group 1 than in Group 2, being the only indicator that carried a statistically significant difference between the groups at baseline. At T2, the mean + SD values for Group 1 were significantly higher than those for Group 2, pointing out an important change. The difference ∆ T1–T2 for Group 2 was also statistically significant, compared to Group 1. The same pattern for BOP evolution was repeated for T3 and T4 (Table 4 and Figure 7), suggesting more relevant changes in Group 2 compared to Group 1. At T5, the BOP mean values + SD for Groups 1 and 2 were quite similar.

**Figure 8:**
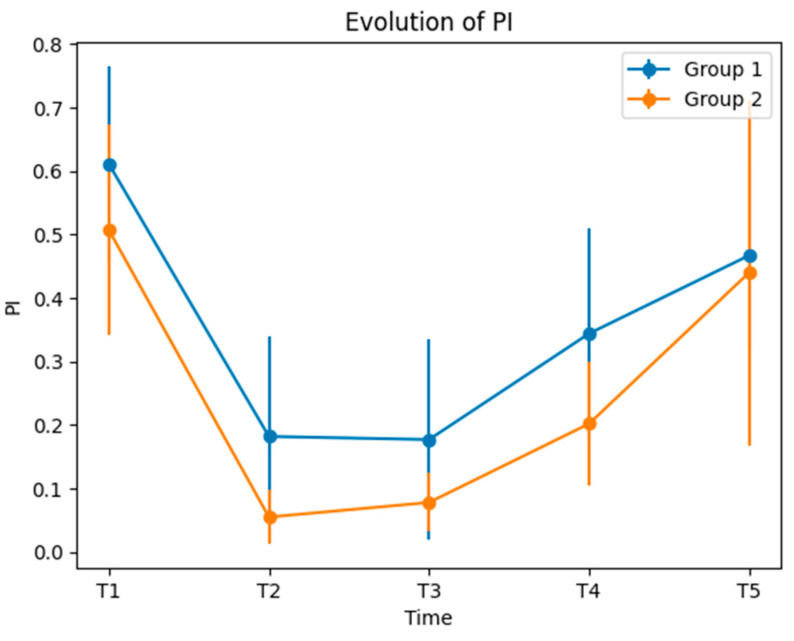
Evolution of PI.

Regarding the dynamics of PI, when entering the study, all patients were instructed to maintain very good oral hygiene. The goal was to maintain a PI value under 0.3, in order to enhance healing. PI values were recorded during the study to track the hygiene performance of the subjects, aiming not to compromise the results by poor oral hygiene. The PI values at T1 for Group 1 and Group 2 showed a marginally significant difference. The lowest PI values were recorded at T2 and T3, with statistical significance between the groups. Similar differences were recorded at T4, but the PI values were higher compared to T3. At T5, the PI values for the two groups were close, with no statistical significance (Table 4 and Figure 8).

**Figure 9:**
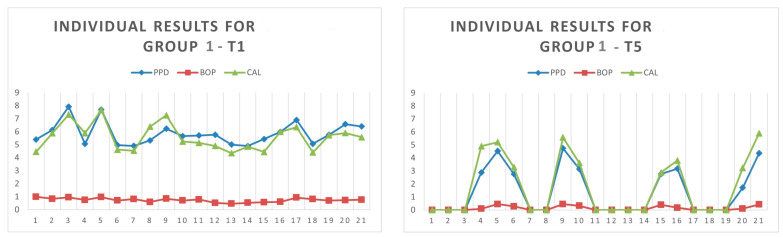
Individual measurement results for Group 1, at T1 and T5.

**Figure 10:**
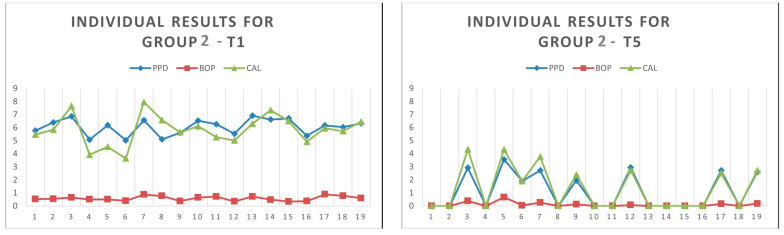
Individual measurement results for Group 2, at T1 and T5.

The graphs below (Figure 9 and Figure 10) show an overview of the individual differences at T1 and T5, for the PPD, CAL and BOP value dynamics. No notable differences between the groups appeared at baseline (T1). The PPD and CAL mean values at T1 were much higher, compared to T5, in both groups. When comparing the PPD and CAL at T5, the values for Group 2 were considerably better than those for Group 1. However, at T5, the PPD mean values for all patients in Group 2 were below 4 mm, which was considered normal. In the cases of PPD, CAL, BOP values recorded on the 0 axis of the graph, complete healing had been previously reached, with the measurements for those patients being no longer recorded at T5 (12 patients out of 21 for Group 1 and 11 patients out of 19 for Group 2). The recorded values for each patient at T1 and T5 are given in Table A1, Table A2, Table A3 and Table A4 (Appendix A).

### 4.2. Network Analysis—Gaussian Graphical Models (GGMs)

After assessing the results obtained from the measurements of the four indicators at T1–T5 time markers, and recording the clinical evolution of the periodontal status, the research was completed with computational modeling. The inferences between the four specific indicators used to measure the success of the laser treatment, namely PPD, CAL, BOP and PI, as well as the linkages between these credentials in time (T1–T5), were further analyzed.

In this respect, network analysis was first implemented through GGMs. Two models were configured and assessed using two distinctive methods, namely the extended Bayesian information criteria with graphical least absolute shrinkage and selection operator (EBICglasso) and partial correlation (PCOR).

**Figure 11:**
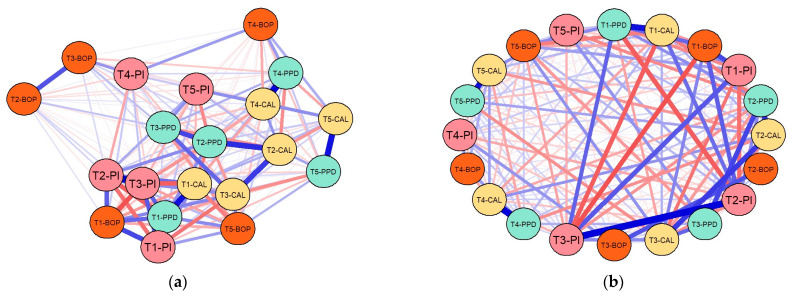
Results of GGMs: (**a**) EBICglasso method; (**b**) PCOR method. Authors*’* design in RStudio 4.1.3.

The graphical models resulting from the estimations are presented in Figure 11.

Strong interlinkages were revealed by both GGMs, with both positive and negative correlations being outlined between specific credentials across time.

Positive correlations were drawn in blue lines, and negative correlations were rendered with red lines. The nodes of each network were captured through the main indicators used to measure the effects of the laser therapy, namely PPD, CAL, BOP and PI, while the time markers were indicated with T1 (baseline), T2, T3, T4 and T5.

There were notable positive inferences between PPD and CAL in all five time-markers, and particularly in T5, but also between BOP and PI, particularly at baseline (T1). However, there were inverse correlations between these credentials when moving from one time-marker to another, thus revealing the decreases in these values after implementing laser therapy, particularly for PPD and BOP. These indicators proved to be essential proxies for the success of laser therapy in periodontitis.

### 4.3. Structural Equation Modeling (SEM)

**Figure 12:**
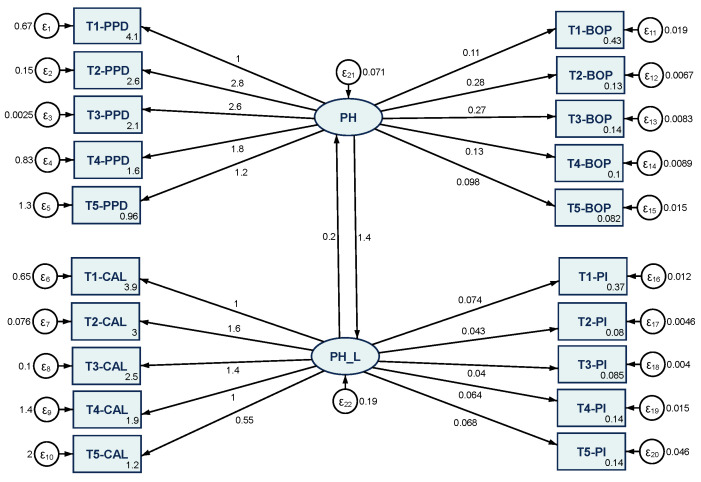
Results of SEM—maximum likelihood estimation method. Source: Authors’ design in RStudio 4.1.3.

Following the results of clinical measurements and network analysis through GGMs, the final step of the research was to determine whether the four indicators captured during the 4-year period were reliable in ensuring periodontal health. In this respect, SEM, which captures periodontal health status as a latent variable (PH, PH_L), reflected and resulting from the indicators, was designed (Figure 12).

The SEM was estimated through the maximum likelihood estimator method and provided robust positive estimated coefficients that were statistically significant at 5% and 1% thresholds.

All estimated coefficients were positive and provided new empirical evidence that the PPD, CAL, BOP, and PI are positive indicators that ensure periodontal health in periodontal laser therapy. Among them, the PPD had the strongest estimations, followed by CAL and BOP, while PI had the least influence, being used as a marker to assess the quality of oral hygiene, its tracking being important, as poor oral hygiene has a negative influence on the treatment results.

## 5. Discussion

Periodontal healing involves the elimination of residual pockets and maintaining a stable periodontal status. However, it is rather difficult to obtain a favorable outcome using solely SRP, as residual pockets exhibiting chronic inflammation are stable and resistant to intervention by non-surgical treatment alone, even after repeated treatment sessions [9], and failed to improve the clinical parameters [20,21,22]. In order to achieve healing, SRP has been tentatively completed by systemic antibiotics, local antiseptics administration [23] or laser debridement.

In fact, one of the major therapeutic challenges in the field of non- surgical periodontal therapy is the use of systemic antibiotics for optimized results, mainly because of the cautious attitude towards their use, in order to limit the development of general microbial resistance [24,25,26,27,28].

On the other hand, local antiseptics, and especially CHX, have proven to be effective in destroying pathogens that cause periodontitis [29], and, thus, are often used in addition to SRP. Previous studies showed that using CHX resulted in less inflammation, improved tissue repair [30], and reduced PPD [31,32]. CHX may be delivered to the periodontal pocket in the form of gel, liquid, and chip [23].

Despite the fact that an ADA 2018 consensus report stated that laser therapy has limited benefits [33], a large number of studies emphasize the better outcome of SRP with laser addition. Minimally invasive laser therapy efficiency is currently widely recognized, showing significant clinical improvement in cases of moderate to advanced periodontal disease [9,34,35], its effects being highlighted both in vitro and in vivo on cell lines of fibroblasts mimicking the periodontal tissue, and up to the mitochondrial level [36,37].

The main focus of the treatment protocol utilized in the present study was to reduce PPD as a clinical indicator and eradicate clinical signs of periodontal disease.

The specific objectives were to reduce the PPD value below 5 mm (4 mm meaning periodontal health), CAL gain, and BOP evaluation, which generally decreases linearly with reducing inflammation and/or healing, and increases in case of poor hygiene, or in case of periodontal disease recurrence. In a high number of cases, BOP represents a major sign of periodontal inflammation, also indicating a certain level of destruction and erosion of the sulcus lining or the ulceration of the sulcular epithelium. PI was assessed as an indicator of oral hygiene, as its lack would certainly result in a compromised treatment outcome.

Taking into account the aforementioned objectives, SRP was supplemented with CHX for Group 1 and laser for Group 2. The analysis of the clinical indicators offers an exhaustive perspective on the evolution of the periodontal status.

The results show a favorable evolution for both groups, in Group 1, prior to T5, 12 out of 21 patients were considered healed, and in Group 2, 11 out of 19 patients reached periodontal health prior to T5 (Figure 9 and Figure 10 and Table A1, Table A2, Table A3 and Table A4). PPD mean values higher than 4 mm at T5 were recorded only in the cases of three patients belonging to Group 1.

The PPD differences between the groups at T1–T5 time points were statistically significant, pointing out broader variations in Group 2 compared to Group 1, with a statistically better outcome in the reduction of the number of residual pockets being recorded for Group 2 (laser) (Table 4).

The significant differences between PPD values at each time point (T2, T3, T4, T5), with consistently lower values for Group 2, showed the greater and consequent impact of the treatment strategy applied to this group, namely laser.

The results of this study reveal that adding laser treatment to SRP significantly decreases PPD, in line with the results of other studies. Laser conditioning of the root surface, de-epithelizing the internal and external area of the pocket, and stimulating bone growth by means of laser energy bring an important benefit in improving periodontal health [35,38,39,40,41,42].

The reduction in PPD was more significant in the laser group in all pocket depth categories (Table 5). Each patient exhibited lower mean PPD values at each evaluation measurement (T1–T5).

The constant reduction in PPD under treatment was shown by the constant shift of the very deep pockets (≤8 mm) to the group of 6–7 mm depth pockets and the 4–5 mm group. The constant shift to a lower depth group was reinforced by the constant reduction in the number of residual pockets following treatment sessions. The laser treatment has shown its superiority in the reduction of the number of residual pockets.

As expected, the PPD decrease was accompanied by CAL gain. The differences between the mean + SD values recorded for Group 1 and 2 were statistically significant for T4 and T5, while for T3, there was a marginal significance (*p* = 0.050). At T2, the differences were notable, but did not reach statistical significance (*p* = 0.263). As shown in Figure 6, the CAL gain was more pronounced at T2 and T3, in both groups, followed by stagnation between T3–T4 (in both groups), and exhibiting a marked growth for Group 2, from T4 to T5, while for Group 1, a slight decrease was observed.

Overall, laser provides beneficial results regarding CAL gain.

Regarding BOP scores, in contrast to PPD and CAL, the BOP scores at T1 showed a statistically significant difference between groups (*p* = 0.004). At T2, T3 and T4, the BOP mean + SD values for Group 1 were higher, with a statistically significant *p* of 0.000. At T5, the BOP values for the two groups did not exhibit a statistically significant difference (*p* = 0.294). Patients exhibited a major reduction in bleeding on probing after treatment, in both groups.

The PI scores showed a marginal statistically significant difference (*p* = 0.051); the differences were statistically significant at T2, T3, and T4, but did not reach statistical significance at T5. However, the PI values at T4 and T5 were higher, for both groups, compared to those recorded at T2 and T3, but still much lower than those recorded at T1.

Favorable outcomes with lasers in periodontal therapy are well-documented in the literature; however, which type of laser is the best for periodontal treatment is still under debate.

A recent study by Nammour et al. aimed to evaluate the efficacy of diode laser, compared to SRP alone. The measurements of PPD, CAL, BOP, and PI were performed at 6, 12, and 18 weeks and 6 months and 1 year, and revealed significant differences between the groups, concluding that laser-assisted SRP resulted in significant clinical improvements. Their findings align with those of the present study [43].

Lazar et al. [44] analyzed the effects of diode laser therapy on the periodontal status of 32 patients for 9 months, and concluded that PI and BOP values notably decreased, while PPD showed only a slight improvement after the laser therapy treatment. Gojkov-Vukelic et al. [45] demonstrated the beneficial effect of the diode laser in chronic periodontitis, on 24 subjects and 1164 periodontal pockets.

The Er:YAG laser is considered a feasible treatment option both in cases of periodontitis and preimplantation, which is another application for non-surgical laser treatment [46,47,48,49,50]. Er:YAG is considered useful as minimally invasive flapless periodontal regenerative surgery during initial therapy [9]. Er,Cr:YSGG laser-assisted conventional periodontal therapy is more effective in reducing oral malodor and improving periodontal healing compared to conventional periodontal therapy alone [6].

A study by Erbil at al. aimed to assess the difference between SRP + Er,Cr:YSGG laser therapy with SRP alone in a group of 59 subjects with advanced periodontitis. PPD, CAL, BOP, and PI were measured at 6 weeks and 3 months. They found significant differences in PPD and BOP at both time points, in favor of the laser group. However, significant differences among groups at any time were recorded for CAL gain [8].

On the contrary, a recent study by Klokkevold et al., carried out on a sample of 15 adults with 90 nonadjacent sites probing ≥ 5 mm, assessed PPD, CAL, BOP, PI, and GR at 1, 3, 6, 9, and 12 month intervals and concluded that adjunctive Er,Cr:YSGG laser therapy with SRP provides clinical improvement in the treatment of moderate-severe periodontitis similar to that of SRP alone and may offer some advantage for deeper (≥7 mm) pockets. This is contrary to our findings: at the 6- and 12-month time points (T2 and T3), the values recorded for all indicators were significantly better for the laser group [51].

Another study, comparing an SRP group with an Er,Cr:YSGG group after 1 year, concluded that both treatment modalities were effective in treating chronic periodontitis, but “the added use of laser may have advantages, particularly in molar tooth sites and deeper pockets” [52].

Another study by the same author, carried out on 49 patients with chronic periodontitis, concluded that the use of Er,Cr:YSGG laser may be of significant clinical benefit. Results collected after 8 ± 3 months revealed significant overall PPD reduction [53]. 

According to Kawamura et al., in terms of the minimal thermal influences during irradiation, less compositional surface change was detected with Er:YAG and Er,Cr:YSGG compared with CO_2_, diode and Nd:YAG lasers [54].

Er,Cr:YSGG laser has been considered to be more effective than diode laser for the treatment of aggressive periodontitis [55].

A recent meta-analysis by Li et al. aimed at systematically assessing all of the available evidence on the clinical effectiveness of Er,Cr:YSGG lasers in the non-surgical treatment of chronic periodontitis. The data were extracted from 16 randomized controlled clinical trials, published between 2000–2020, comparing the effectiveness of SRP + Er,Cr:YSGG laser with SRP alone. The study emphasized that Er,Cr:YSGG provided additional effectiveness in PPD reduction and CAL gain at short-term follow-ups [56].

The significant differences found in the indicators’ evolution at different time points emphasize the importance of long-term monitoring, which was accomplished in the present study, carried out over a 4-year period.

The beneficial effects of laser therapy are more than evident according to previous studies, and the literature on the subject is improving on a daily basis, covering extensive research in the field of periodontal therapy. To our knowledge, a limited number of studies involving computational prediction and network analysis in the medical field are currently available. However, this is considered a potentially effective estimation tool, enabling data systematization, and suggesting successful therapeutic algorithms for practitioners.

The empirical research configured in the current scientific paper was based on two advanced approaches of data modeling, namely GGMs and SEM.

GGMs are used to estimate a network of correlations between variables, based on data sets [57].

Using visual rendering, GGMs provide overall insights of the causal dependency and intensity of interactions among variables, being considered efficient in analyzing complex datasets with multiple variables, including medical applications such as omics [16,58], body-fluids proteomes [59], public health [19], COVID-19 epidemiological assessment [60], and characterization of rare diseases (Prader–Willi Syndrome) [18].

In the GGMs network model, the observed variables are represented with nodes, which are connected with an edge if two variables are not independent after conditioning on all other observed variables.

An undirected network of partial correlation coefficients (both positive and negative) is involved in GGMs, which is reflected graphically in the width and saturation of the edges between the nodes, making it a network model of conditional associations and preventing spurious correlation [18]. A variation–covariance matrix was used in this approach to determine how variables were related to each other and the direct and indirect effects of one variable on another.

The GGMs resulting from processing the data collected in this study (Figure 11) revealed strong interlinkages, with both positive and negative correlations. Positive correlations were rendered with blue lines, and negative correlations were rendered with red lines, connecting the nodes that, in our case, were represented by the indicators used to measure the effects of the laser therapy, namely PPD, CAL, BOP and PI, at different time points: T1, T2, T3, T4 and T5. According to GGMs, positive inferences between PPD and CAL were noticed at all five time points, and particularly at T5, while positive interferences between BOP and PI were particularly noticeable at baseline (T1). The inverse correlations between these indicators, when moving from one time point to another, revealed decreases in their values as a result of laser therapy, being particularly noticeable for PPD and BOP.

SEM, a flexible multivariate technique that allows examination of relationships among variables, has been used in medicine as well: e.g., in epidemiology [61], cardiology [62], and public health [63].

SEM, which combines confirmatory factors, path analysis, and regression, was used to enhance the robustness of the results and the methodological rationale. The SEM was estimated through the maximum likelihood estimator method and provided robust positive estimated coefficients, allowing us to determine how each variable depended upon its immediate causal predecessors, and determining if PPD, CAL, BOP and PI, captured during the 4-year period, were reliable in ensuring periodontal health. In Figure 12, SEM captures periodontal health status as a latent variable (PH, PH_L), reflected and resulting from the indicators, reflecting the configuration of the cause effect hypothesis. The effect of one variable on another is represented by →, and estimated from the sample data.

Two components were present in the SEM models, namely a measurement model and a structural model, which defined causal and association relations between the measured constructs. The SEM-estimated coefficients were all positive and offer new empirical evidence to support that PPD, CAL, BOP, and PI are positive indicators that ensure periodontal health after periodontal laser therapy. Of these, PPD had the strongest estimates, followed by CAL and BOP, while PI had the least influence power, being used as a marker for assessing the quality of oral hygiene, tracing it as important, as poor oral hygiene has a negative influence on the results of treatment.

The advantage of both methods resides in the identification of direct and indirect relationships between one variable and another [18,60]. The GGMs approach allowed us to design the model of the whole system constructed by fitting a sub-model sequence, whereas the SEM approach provided a unitary setting through a single model for the whole system of variables under investigation in the current study.

## 6. Conclusions

The SRP + Er,Cr:YSGG laser periodontal treatment protocol demonstrated its efficiency, by showing significant clinical improvements, and provided a better clinical outcome, after 4 years of treatment, compared to SRP + CHX. Furthermore, significant differences between the groups were observed at 6-month, 1-year, and 2-year follow-ups, clearly in favor of the laser group. PPD, CAL, BOP, and PI proved to be essential proxies for the success of periodontal therapy, as shown by GGMs and SEM. GGMs and SEM were relevant to the study since they both originated from path analysis and provided comprehensive graphical representations of the associations between variables, ordered temporally.

## Figures and Tables

**Table 1:** Group characteristics.

Group	Group 1 (CHX)	Group 2 (Laser)
No. of patients at baseline	21	19
Gender M/F, %	9/12, 42%/58%	9/10, 47%/53%
Age (mean ± SD)	51.38 ± 8.737	51.631 ± 8.200
Smokers/Nonsmokers	10/11	13/6
Stage of periodontitis 2/3/4	9/12/0	8/11/0

**Table 2:** Er,Cr:YSGG laser parameters.

Laser	Power Average (W)	Frequency (Hz)	Pulse Duration (µs)	Pulse Energy (mJ)	Power Peak (W)	Time (s)
Er,Cr:YSGG 2780 nm 14 mm glass tip	2	20	60	2 W/20 Hz = 100 mJ	1000 W 100 mJ/60 µs = 1666 J/s	10 s/mm depth monorooted teeth 15 s/mm depth multirooted teeth

**Table 3:** Number of patients in each group, at the different time markers.

Time	Group 1 (CHX)	Group 2 (Laser)
Baseline (T1)	21	19
T2	19	19
T3	19	19
T4	16	15
T5	9	8

**Table 4:** Clinical parameters (mean + SD) and group comparison.

Indicator	Time	Group 1 (CHX) Mean ± SD	Group 2 (Laser) Mean ± SD	Group Comparison *p*
PPD	Baseline (T1)	5.850 ± 0.869	6.062 ± 0.615	0.375
	T2	4.276 ± 0.837	3.199 ± 0.828	0.000 *
	T3	3.762 ± 0.808	2.398 ± 0.333	0.000 *
	T4	3.473 ± 0.705	2.466 ± 0.288	0.000 *
	T5	3.344 ± 1.000	2.664 ± 0.504	0.008 *
	∆ T1–T2	1.573 ± 0.604	2.863 ± 0.842	0.000 *
	∆ T1–T3	2.087 ± 0.572	3.663 ± 0.665	0.000 *
	∆ T1–T4	2.377 ± 0.669	3.596 ± 0.607	0.000 *
	∆ T1–T5	2.506 ± 0.975	3.397 ± 0.548	0.000 *
CAL	Baseline (T1)	−5.568 ± 1.016	−5.838 ± 1.150	0.438
	T2	−4.600 ± 1.194	−4.196 ± 1.059	0.263
	T3	−4.088 ± 1.191	−3.357 ± 1.095	0.050 ^†^
	T4	−4.092 ± 1.192	−3.368 ± 1.003	0.043 *
	T5	−4.266 ± 1.132	−3.084 ± 0.927	0.000 *
	∆ T1–T2	−0.968 ± 0.677	−1.642 ± 0.818	0.007 *
	∆ T1–T3	−1.479 ± 0.720	−2.481 ± 0.734	0.000 *
	∆ T1–T4	−1.475 ± 0.826	−2.470 ± 0.721	0.000 *
	∆ T1–T5	−1.302 ± 0.817	−2.75 ± 0.894	0.000 *
BOP	Baseline (T1)	0.744 ± 0.154	0.587 ± 0.176	0.004 *
	T2	0.334 ± 0.157	0.055 ± 0.048	0.000 *
	T3	0.359 ± 0.158	0.054 ± 0.046	0.000 *
	T4	0.287 ± 0.166	0.119 ± 0.072	0.000 *
	T5	0.296 ± 0.145	0.236 ± 0.204	0.294
	∆ T1–T2	0.410 ± 0.144	0.531 ± 0.158	0.015 *
	∆ T1–T3	0.385 ± 0.169	0.532 ± 0.152	0.006 *
	∆ T1–T4	0.457 ± 0.221	0.467 ± 0.180	0.875
	∆ T1–T5	0.447 ± 0.176	0.350 ± 0.236	0.152
PI	Baseline (T1)	0.611 ± 0.160	0.507 ± 0.166	0.051
	T2	0.182 ± 0.128	0.055 ± 0.042	0.000 *
	T3	0.177 ± 0.130	0.078 ± 0.046	0.002 *
	T4	0.344 ± 0.149	0.202 ± 0.098	0.000 *
	T5	0.467 ± 0.273	0.440 ± 0.272	0.756
	∆ T1–T2	0.429 ± 0.187	0.452 ± 0.152	0.670
	∆ T1–T3	0.433 ± 0.179	0.429 ± 0.159	0.940
	∆ T1–T4	0.267 ± 0.239	0.305 ± 0.179	0.570
	∆ T1–T5	0.144 ± 0.305	0.067 ± 0.389	0.493

* Statistically significant value (*p* < 0.05) ^†^ Borderline statistically significant value (*p* = 0.05). ∆ Difference between indicator values at certain time points.

**Table 5:** The number of PPD sites regarding their depth at different time points.

PPD Depth		Group 1 (CHX)	Group 2 (Laser)
**4–5 mm**	Baseline (T1)	1155	946
	T2	1167	765
	T3	1061	261
	T4	688	183
	T5	304	195
**6–7 mm**	Baseline (T1)	763	890
	T2	493	161
	T3	290	34
	T4	234	7
	T5	140	6
**≥8 mm**	Baseline (T1)	413	423
	T2	107	21
	T3	67	11
	T4	44	6
	T5	32	0

## Data Availability

The dataset can be provided by the authors upon request.

## Appendix A

**Table A1:** Mean values of PPD, CAL, and BOP for each patient belonging to Group 1, at T1.

Patient (*n* = 21)	PPD	BOP	CAL
1	5.402439024	1	4.457831325
2	6.130841121	0.841121495	5.887850467
3	7.932330827	0.954887218	7.315789474
4	5.067307692	0.75	5.894230769
5	7.692810458	0.967320261	7.666666667
6	4.964285714	0.705357143	4.616071429
7	4.905882353	0.823529412	4.541176471
8	5.333333333	0.594594595	6.387387387
9	6.233082707	0.850746269	7.268656716
10	5.666666667	0.708333333	5.25
11	5.708333333	0.791666667	5.145833333
12	5.77037037	0.525925926	4.903703704
13	5.0125	0.4625	4.35
14	4.901234568	0.530864198	4.851851852
15	5.435643564	0.574257426	4.445544554
16	5.984496124	0.604651163	5.984496124
17	6.901098901	0.934065934	6.351648352
18	5.071428571	0.80952381	4.404761905
19	5.77037037	0.696296296	5.733333333
20	6.581967213	0.73553719	5.901639344
21	6.401459854	0.773722628	5.576642336

**Table A2:** Mean values of PPD, CAL, and BOP for each patient belonging to Group 1, at T5.

Patient (*n* = 21)	PPD	BOP	CAL
1	0	0	0
2	0	0	0
3	0	0	0
4	2.891304348	0.094202899	4.905797101
5	4.533333333	0.453333333	5.213333333
6	2.753623188	0.275362319	3.282608696
7	0	0	0
8	0	0	0
9	4.746031746	0.446969697	5.579365079
10	3.148809524	0.31547619	3.607142857
11	0	0	0
12	0	0	0
13	0	0	0
14	0	0	0
15	2.775641026	0.397435897	2.891025641
16	3.172839506	0.166666667	3.802469136
17	0	0	0
18	0	0	0
19	0	0	0
20	1.714285714	0.101190476	3.226190476
21	4.361111111	0.41958042	5.888888889

**Table A3:** Mean values of PPD, CAL, and BOP for each patient belonging to Group 2, at T1.

Patient (*n* = 19)	PPD	BOP	CAL
1	5.774193548	0.532258065	5.475806452
2	6.409090909	0.554545455	5.854545455
3	6.871559633	0.651376147	7.651376147
4	5.086538462	0.514285714	3.933333333
5	6.188405797	0.514492754	4.536231884
6	5.033333333	0.4	3.65
7	6.570469799	0.879194631	7.959731544
8	5.10989011	0.769230769	6.582417582
9	5.605633803	0.38028169	5.647887324
10	6.538461538	0.647435897	6.115384615
11	6.272727273	0.727272727	5.272727273
12	5.54109589	0.356164384	5.020547945
13	6.92	0.733333333	6.306666667
14	6.626760563	0.485915493	7.338028169
15	6.712418301	0.346405229	6.509803922
16	5.380952381	0.375	4.928571429
17	6.172932331	0.892307692	5.954887218
18	6.045045045	0.783783784	5.738738739
19	6.333333333	0.611111111	6.456790123

**Table A4:** Mean values of PPD, CAL, and BOP for each patient belonging to Group 2, at T5.

Patient (*n* = 19)	PPD	BOP	CAL
1	0	0	0
2	0	0	0
3	2.920634921	0.380952381	4.333333333
4	0	0	0
5	3.56	0.666666667	4.32
6	1.901234568	0.043209877	1.895061728
7	2.712121212	0.257575758	3.772727273
8	0	0	0
9	1.955555556	0.127777778	2.411111111
10	0	0	0
11	0	0	0
12	2.946428571	0.06547619	2.720238095
13	0	0	0
14	0	0	0
15	0	0	0
16	0	0	0
17	2.724137931	0.166666667	2.510204082
18	0	0	0
19	2.598765432	0.185185185	2.716049383
